# Incidence and Outcomes of Acute Kidney Injury Requiring Renal Replacement Therapy in Patients on Percutaneous Mechanical Circulatory Support with Impella-CP for Cardiogenic Shock

**DOI:** 10.7759/cureus.6591

**Published:** 2020-01-07

**Authors:** Fadi Fahad, Muhammad Hamza Saad Shaukat, Neil Yager

**Affiliations:** 1 Cardiology, Albany Medical College, Albany, USA; 2 Internal Medicine, Albany Medical College, Albany, USA

**Keywords:** cardiogenic shock, acute kidney injury, renal replacement therapy, mechanical circulatory support

## Abstract

Background: Acute kidney injury (AKI) complicating cardiogenic shock is associated with increased mortality. We hypothesize that renal replacement therapy (RRT) improves survival in cardiogenic shock supported by Impella-CP (Abiomed, Danvers, MA) complicated by AKI.

Methods: A retrospective chart review identified 34 patients on Impella-CP for cardiogenic shock between January 2015 and December 2017. AKI was defined as an increase in serum creatinine≥0.3 mg/dL from baseline. Three groups were analyzed: AKI plus RRT, AKI minus RRT, and no AKI. Pre-existing dialysis patients were excluded. The only indication for RRT was AKI not responding to diuretics. Thirty-day mortality was analyzed.

Results: There were 13 patients with no AKI, 9 with AKI plus RRT groups, and 12 with AKI minus RRT. Thirty-day mortality was similar between no AKI and AKI plus RRT groups [30.8% (4/13) vs.22.2% (2/9), p=0.48; relative risk [RR] 2.25 (95% confidence interval [CI] 0.22-22.1)]. Thirty-day mortality was higher in AKI minus RRT group compared to the no AKI group [75.0% (9/12) vs. 30.8% (4/13); p=0.03; RR 6.75 (95% CI 1.16-39.2)].

Conclusion: In cardiogenic shock patients on Impella-CP, AKI minus RRT is associated with a higher 30-day mortality compared to patients without AKI and/or patients with AKI plus RRT. Short-term mortality may improve in cardiogenic shock patients with AKI who are treated with RRT.

## Introduction

Despite advances in technology for the treatment of cardiogenic shock, mortality has not dramatically improved [1]. Cardiogenic shock primarily occurs in the setting of acute myocardial infarction. Standard of care in acute myocardial infraction complicated by cardiogenic shock is revascularization [2]. Percutaneous mechanical circulatory support devices, such as the Impella-CP (Abiomed, Danvers, MA), are used as supportive therapy in cardiogenic shock, despite limited randomized clinical data [3]. Registry data do suggest improved outcomes in cardiogenic shock supported by the Impella-CP [4]. However, there is a paucity of data surrounding outcomes in cardiogenic shock patients with concurrent acute kidney injury (AKI), managed with or without renal replacement therapy (RRT). Cardiogenic shock complicated by AKI requiring RRT is associated with increased mortality [5,6]. We hypothesize that early RRT for AKI in cardiogenic shock patients on Impella-CP improves survival.

## Materials and methods

Our cohort was a single-center retrospective study including all patients on Impella-CP for cardiogenic shock admitted to Albany Medical Center between January 2015 and December 2017. Data were obtained by a retrospective review of the electronic medical record. Cardiogenic shock was defined as elevated serum lactate (>2.0 mmol/L) and hypotension requiring inotrope/vasopressors to maintain a mean arterial blood pressure above 65 mmHg. Eligible patients were classified based on AKI at presentation (increase in serum creatinine>0.3 mg/dL from baseline); those with AKI were further categorized by RRT. This led to three groups: no AKI, AKI minus RRT, and AKI plus RRT. RRT included hemodialysis or continuous RRT.

The exclusion criteria included pre-existing hemodialysis-dependent patients, unknown baseline renal function, or patients lost to follow-up within 30 days. The chi-square test was utilized to compare 30-day mortality of AKI plus RRT and no AKI groups as well as AKI minus RRT and no AKI groups. Continuous variables (lab parameters at presentation) were compared between groups using ANOVA. Type 1 error was prespecified at less than or equal to 5%. The protocol was approved by the Institutional Review Board at Albany Medical Center.

## Results

Between January 2015 and December 2017, 34 patients with cardiogenic shock on Impella-CP met the inclusion criteria for our study. There were 13 patients with no AKI, 9 had AKI plus RRT, and 12 had AKI minus RRT. The only indication for RRT was AKI not responding to diuretics (oliguria or anuria). Baseline characteristics and lab parameters at presentation are included in Tables 1, 2.

**Table 1:** Lab parameters at presentation (±SEM) SEM, standard error of the mean; AKI, acute kidney injury; RRT, renal replacement therapy; INR, international normalized ratio. *Renal replacement therapy (hemodialysis or continuous renal replacement therapy).

	no-AKI	AKI without RRT*	AKI+RRT*	p-value
Hemoglobin (g/dL)	13.2±0.529	12.1±0.583	12.9±1.46	0.448
Serum creatinine (mg/dL)	0.972±0.071	2.10±0.320	2.55±0.918	0.010
Serum lactate (mmol/L)	5.82±4.01	4.73±0.628	4.75±0.329	0.904
Serum HCO_3_ (mEq/L)	20.9±1.42	19.7±1.40	19.7±2.78	0.773
INR	1.96±0.529	1.70±0.416	1.67±0.509	0.929
Arterial blood gas				
pH	7.20±0.053	7.28±0.024	7.18±0.055	0.466
pCO_2_	47.0±5.24	41.8±2.63	51.0±9.43	0.687
pO_2_	146±46.9	163±41.2	95.2±18.4	0.526

**Table 2:** Baseline characteristics AKI, acute kidney injury; HTN, hypertension; DM, diabetes mellitus; CVA/TIA, cerebrovascular accident/transient ischemic attack; GFR, glomerular filtration rate; PCI, percutaneous coronary intervention; ACEi/ARB, angiotensin-converting enzyme inhibitor/angiotensin receptor blocker; NSAID, non-steroidal anti-inflammatory drug.

	Cardiogenic shock with AKI n=21 (%)	Cardiogenic shock without AKI n=13 (%)	p-value
Age (years±SD)	60.7±14.5	63.3±11.8	0.556
Female	2 (9.52)	3 (23.1)	0.455
HTN	15 (71.4)	8 (61.5)	0.428
DM	5 (23.8)	3 (23.1)	0.858
Dyslipidemia	11 (52.4)	7 (53.8)	0.985
CVA/TIA	3 (14.3)	2 (15.4)	0.781
GFR<60 mL/min/1.73 m^2^	4 (19.0)	1 (7.69)	0.211
Anemia (Hb<11 g/dL)	7 (33.3)	0 (0)	0.008
Atrial fibrillation	4 (19.0)	2 (15.4)	0.873
Peripheral vascular disease	4 (19.0)	3 (23.1)	0.841
Valvular heart disease	5 (23.8)	2 (15.4)	0.497
Coronary artery disease	9 (42.9)	5 (38.5)	0.790
Prior PCI	7 (33.3)	6 (46.2)	0.459
Tobacco use in prior 12 months	7 (33.3)	5 (38.5)	0.825
Medication use prior to presentation			
ACEi/ARB	10 (47.6)	6 (46.2)	0.985
Beta blocker	11 (52.4)	5 (38.5)	0.515
Calcium channel blocker	0 (0)	3 (23.1)	0.044
Diuretics	10 (47.6)	4 (30.8)	0.341
Aspirin	11 (52.4)	5 (38.5)	0.515
NSAID	1 (4.76)	0 (0)	0.283
Digoxin	0 (0)	1 (7.69)	0.168
Statin	12 (57.1)	5 (38.5)	0.316
Metformin	2 (9.52)	3 (23.1)	0.455

AKI was associated with higher 30-day mortality compared to patients with no AKI in our cardiogenic shock cohort [52.4% (11/21) vs. 30.8% (4/13), p=0.01; relative risk [RR] 6.16 (95% confidence interval [CI] 1.41-26.7)]. Thirty-day mortality was similar in patients with no AKI and AKI plus RRT [30.8% (4/13) vs. 22.2% (2/9), p=0.48; RR 2.25 (95% CI 0.22-22.1)]. Patients with AKI minus RRT had a higher 30-day mortality than patients with no AKI [75.0% (9/12) vs. 30.8% (4/13); p=0.03; RR 6.75 (95% CI 1.16-39.2)]. Outpatient use of calcium channel blockers and hemoglobin greater than 11 g/dL were associated with decreased likelihood of AKI at presentation (Table 1). Serum creatinine at presentation was higher in patients who received RRT for AKI; serum lactic acid, hemoglobin, and arterial pH were similar across all groups (Table 2).

## Discussion

AKI in severe system illness and particularly cardiogenic shock is associated with poor outcomes [7,8]. AKI in cardiogenic shock has been postulated to be caused by venous congestion and reduced renal perfusion [9]. 

Our single-center retrospective study evaluated outcomes of cardiogenic shock in patients on Impella-CP complicated by AKI with and without RRT. We found that patients with AKI had a similar 30-day mortality when receiving RRT compared to patients without AKI at presentation.

It seems intuitive that early recognition of AKI in cardiogenic shock and aggressive treatment with RRT may improve outcomes, but data are conflicting. Aside from cardiogenic shock populations, the Impella-CP device is shown to be protective against AKI in patients undergoing high-risk percutaneous coronary intervention [10]. In a single-center study, patients on extracorporeal membrane oxygenation for cardiogenic shock with associated AKI did not have improved short-term survival with RRT [11]. Whether this cohort was more critically ill than the patients we studied is not known.

The limitations of our study are acknowledged: small sample size, retrospective examination, single-center review, and wide confidence intervals.

## Conclusions

AKI is an independent predictor of mortality in cardiogenic shock. Based on our single-center, retrospective comparison of cardiogenic shock supported by Impella-CP, 30-day mortality was similar between patients with no AKI and those who received RRT for AKI. Early initiation of RRT for AKI coupled with improved left ventricular unloading with Impella-CP may offset the detrimental effects of AKI in cardiogenic shock. Our study provides preliminary evidence to warrant further investigation on early RRT for AKI in cardiogenic patients supported with Impella-CP.

## References

[1] van Diepen S, Katz JN, Albert NM (2017). Contemporary management of cardiogenic shock: a scientific statement from the american heart association. Circulation.

[2] Hochman JS, Sleeper LA, Webb JG (1999). Early revascularization in acute myocardial infarction complicated by cardiogenic shock. SHOCK Investigators. Should we emergently revascularize occluded coronaries for cardiogenic shock.. N Engl J Med.

[3] Ouweneel DM, Eriksen E, Sjauw KD (2017). Percutaneous mechanical circulatory support versus intra-aortic balloon pump in cardiogenic shock after acute myocardial infarction. J Am Coll Cardiol.

[4] O'Neill WW, Grines C, Schreiber T (2018). Analysis of outcomes for 15,259 US patients with acute myocardial infarction cardiogenic shock (AMICS) supported with the Impella device. Am Heart J.

[5] Inampudi C, Adegbala O, Akintoye E, Alvarez P, Briasoulis A (2019). Characteristics and outcomes of patients with cardiogenic shock complicated by acute kidney injury requiring hemodialysis. J Heart Lung Transplant.

[6] Marenzi G, Cosentino N, Marinetti A (2017). Renal replacement therapy in patients with acute myocardial infarction: rate of use, clinical predictors and relationship with in-hospital mortality. Int J Cardiol.

[7] Rewa O, Bagshaw SM (2014). Acute kidney injury-epidemiology, outcomes and economics. Nat Rev Nephrol.

[8] Tarvasmäki T, Haapio M, Mebazaa A Acute kidney injury in cardiogenic shock: definitions, incidence, haemodynamic alteration, and mortality. Eur J Heart Fail.

[9] Damman K, Testani JM (2015). The kidney in heart failure: an update. Eur Heart J.

[10] Flaherty MP, Pant S, Patel SV (2017). Hemodynamic support with a microaxial percutaneous left ventricular assist device (Impella) protects against acute kidney injury in patients undergoing high-risk percutaneous coronary intervention. Circ Res.

[11] Lin YC, Lin YC, Lin FY (2014). Early continuous renal replacement therapy in cardiogenic shock patients with severe acute kidney injury undergoing extracorporeal membrane oxygenation. Cardiorenal Med.

